# Supplementary material for: Effect of Marinating in Dairy-Fermented Products and Sous-Vide Cooking on the Protein Profile and Sensory Quality of Pork Longissimus Muscle
Source: Foods. 2023 Aug 30;12(17):3257. doi: 10.3390/foods12173257 (PMC10486606; doi:10.3390/foods12173257)
Supplement: Supplementary file 1 [file foods-12-03257-s001.zip › foods-2574831-supplementary.pdf]

Table S1. The results of quantitative densitometric analysis after SDS-PAGE electrophoresis (percentage of the band in the lane).

Marinating type	Marinating time [days]	Sous-vide temp. [°C]	nebulin/titin and myosin (HC)	calpains *m* and μ	actin	myosin (LC3)
Control	3	raw	15.27±0.46^{J}	1.09±0.08^{CDE}	15.08±0.18^{K}	0.46±0.11^{AB}
		60	10.48±0.13^{H}	0.86±0.11^{ABCD}	12.36±0.36^{HI}	1.79±0.16^{E}
		80	5.63±0.2^{BC}	0.51±0.12^{AB}	9.61±0.21^{CD}	1.48±0.10^{D}
	6	raw	12.94±0.24^{I}	0.91±0.16^{ABCD}	13.87±0.19^{J}	0.68±0.07^{B}
		60	10.01±0.16^{GH}	0.81±0.19^{ABC}	8.44±0.34^{C}	2.34±0.09^{F}
		80	4.03±0.1^{A}	0.45±0.06^{A}	6.44±0.42^{AB}	2.98±0.11^{GH}
Yoghurt	3	raw	17.75±0.27^{L}	2.52±0.13^{L}	15.80±0.60^{KL}	0.28±0.04^{A}
		60	10.80±0.26^{H}	1.45±0.12^{EFGH}	8.64±0.26^{C}	1.85±0.12^{E}
		80	3.65±0.15^{A}	0.93±0.14^{BCD}	6.59±0.15^{B}	1.98±0.05^{E}
	6	raw	16.23±0.35^{K}	2.36±0.16^{KL}	15.72±0.37^{KL}	1.03±0.02^{C}
		60	8.94±0.39^{EF}	1.20±0.15^{CDEF}	11.31±0.31^{FGH}	2.75±0.04^{G}
		80	3.63±0.20^{A}	0.93±0.11^{BCD}	11.02±0.45^{EFG}	3.08±0.02^{HI}
Kefir	3	raw	17.89±0.6^{L}	2.21±0.17^{JKL}	15.84±0.22^{KL}	0.46±0.05^{AB}
		60	9.55±0.44^{FG}	1.79±0.2^{GHIJ}	10.05±0.49^{DE}	1.82±0.04^{E}
		80	5.11±0.18^{B}	1.10±0.11^{CDE}	5.32±0.45^{A}	2.00±0.07^{E}
	6	raw	15.93±0.22^{JK}	2.15±0.27^{IJKL}	16.60±0.08^{L}	1.17±0.06^{C}
		60	8.24±0.17^{E}	1.92±0.17^{HIJK}	13.26±0.31^{IJ}	2.98±0.09^{GH}
		80	5.75±0.12^{BCD}	0.96±0.12^{BCD}	11.36±0.36^{FGH}	3.25±0.09^{I}
Buttermilk	3	raw	16.55±0.48^{K}	2.59±0.23^{L}	15.23±0.54^{K}	0.45±0.06^{AB}
		60	10.09±0.09^{GH}	1.73±0.17^{GHI}	13.24±0.46^{IJ}	0.52±0.10^{AB}
		80	6.43±0.22^{CD}	1.33±0.10^{DEFG}	11.96±0.48^{GH}	2.36±0.13^{F}
	6	raw	16.14±0.18^{JK}	2.54±0.13^{L}	11.95±0.53^{GH}	1.47±0.08^{D}
		60	9.29±0.25^{FG}	1.61±0.09^{FGH}	10.28±0.21^{DEF}	2.84±0.10^{GH}
		80	6.60± 0.25^{D}	1.16±0.14^{CDEF}	8.70±0.54^{C}	2.93±0.07^{GH}
SEM			0.081	0.023	0.145	0.007
Effect of:						
Marinating type			***	***	***	***
Marinating time			***	*	NS	***
Sous-vide temperature			***	***	***	***
Marinating type x Marinating time			***	NS	***	***
Marinating type x SV temperature			***	***	***	***
Marinating time x SV temperature			***	NS	***	***
Marinating type x and time x SV temperature			***	NS	***	***

Mean ±SD; HC – myosin heavy chains; LC3 – myosin light chain; (A, B, …) - different letters in column indicate significant differences between means for samples marinated in liquid fermented dairy products (FDP) and control sample (HSD test: $p \le 0.05$); * = $p \le 0.05$; ** = ***= $p \le 0.001$; NS - non-significant effect = $p > 0.05$.

Table S2. Effect of marinating and SV cooking on pork sensory attribute: color and texture.

Marinating		SV temp [°C]	Color	Uniformity of color	Hardness	Tenderness	Adhesiveness to teeth	Juiciness
Type	Time [days]							
Control	3	60	2.52 ±0.08^{A}	4.03 ±0.05^{A}	4.53 ±0.08^{D}	4.80 ±0.09^{I}	3.95 ±0.05^{CDEF}	4.03 ±0.08^{F}
		80	3.72 ±0.08^{E}	4.55 ±0.10^{C}	4.02 ±0.08^{C}	1.95 ±0.10^{A}	4.95 ±0.05^{G}	1.97 ±0.08^{E}
	6	60	3.17 ±0.05^{CD}	3.97 ±0.05^{A}	4.53 ±0.12^{D}	4.78 ±0.08^{I}	4.02 ±0.10^{EF}	4.05 ±0.05^{FG}
		80	3.88 ±0.08^{FGH}	4.95 ±0.05^{E}	3.82 ±0.08^{B}	2.07 ±0.12^{AB}	3.85 ±0.05^{CDE}	1.85 ±0.10^{DE}
Yoghurt	3	60	2.97 ±0.10^{B}	3.98 ±0.10^{A}	4.77 ±0.05^{E}	4.78 ±0.04^{I}	4.00 ±0.06^{DEF}	4.52 ±0.13^{I}
		80	3.92 ±0.08^{GH}	4.95 ±0.05^{E}	3.58 ±0.17^{A}	2.83 ±0.08^{G}	3.93 ±0.10^{CDEF}	1.55 ±0.08^{BC}
	6	60	3.28 ±0.08^{D}	4.27 ±0.08^{B}	4.83 ±0.08^{E}	4.05 ±0.08^{H}	3.83 ±0.10^{BCD}	4.22 ±0.08^{GH}
		80	3.92 ±0.08^{GH}	4.98 ±0.04^{E}	3.48 ±0.08^{A}	2.50 ±0.09^{EF}	3.62 ±0.12^{A}	1.30 ±0.06^{A}
Kefir	3	60	3.25 ±0.05^{D}	4.53 ±0.08^{C}	4.78 ±0.12^{E}	4.80 ±0.06^{I}	4.00 ±0.09^{DEF}	4.30 ±0.06^{H}
		80	3.75 ±0.10^{EF}	4.78 ±0.08^{D}	3.53 ±0.10^{A}	2.18 ±0.13^{BC}	4.83 ±0.05^{G}	1.80 ±0.09^{DE}
	6	60	3.98 ±0.08^{H}	4.77 ±0.08^{D}	4.82 ±0.08^{E}	3.93 ±0.12^{H}	4.08 ±0.08^{F}	4.10 ±0.09^{FG}
		80	4.00 ±0.09^{H}	4.97 ±0.05^{E}	3.43 ±0.08^{A}	2.28 ±0.13^{CD}	3.95 ±0.10^{CDEF}	1.68 ±0.08^{CD}
Butter milk	3	60	3.08 ±0.08^{BC}	4.03 ±0.05^{A}	4.82 ±0.08^{E}	4.85 ±0.05^{I}	3.95 ±0.05^{CDEF}	4.62 ±0.08^{I}
		80	3.78 ±0.08^{EFG}	4.97 ±0.05^{E}	3.40 ±0.13^{A}	2.62 ±0.12^{F}	3.78 ±0.10^{ABC}	1.40 ±0.09^{AB}
	6	60	3.67 ±0.08^{E}	4.77 ±0.08^{D}	4.80 ±0.09^{E}	4.90 ±0.09^{I}	4.03 ±0.05^{F}	4.48 ±0.10^{I}
		80	3.80 ±0.06^{EFG}	4.97 ±0.05^{E}	3.53 ±0.08^{A}	2.42 ±0.08^{DE}	3.67 ±0.15^{AB}	1.28 ±0.10^{A}
		SEM	0.006	0.005	0.009	0.009	0.008	0.0075
Effect of:								
Marinating type			***	***	**	***	***	**
Marinating time			***	***	NS	***	***	***
SV temperature			***	***	***	***	***	***
Marinating type x Marinating time			***	***	NS	***	***	***
Marinating type x SV temperature			***	***	***	***	***	***
Marinating time x SV temperature			***	***	*	***	***	NS
Marinating type x Marinating time x SV temperature			**	***	**	***	***	NS

Mean± SD; (A, B, …) - different letters in rows indicate significant differences between means for samples marinated in liquid fermented dairy products (FDP) and control sample (HSD test: $p \le 0.05$); * = $p \le 0.05$; ** = $p \le 0.01$; *** = $p \le 0.001$; NS - non-significant effect = $p > 0.05$.

Table S3. Effect of marinating and SV cooking on pork sensory attribute: odor and flavor

Marinating		SV temp [°C]	Total odour	Intensity of					
Type	Time [days]			sour odour	cooked meat odour	sour flavour	salty flavour	cooked meat flavour	other flavour
Control	3	60	5.00 ±0.00^{G}	1.00 ±0.00^{A}	4.00 ±0.06^{D}	1.00 ±0.00^{A}	1.03 ±0.05^{A}	4.02 ±0.08^{FG}	1.00 ±0.00^{A}
		80	3.98 ±0.08^{CD}	1.00 ±0.00^{A}	4.97 ±0.05^{H}	1.00 ±0.00^{A}	1.02 ±0.04^{A}	4.77 ±0.10^{IJ}	1.20 ±0.06^{CD}
	6	60	4.03 ±0.08^{DE}	1.00 ±0.00^{A}	2.95 ±0.10^{A}	1.00 ±0.00^{A}	1.03 ±0.05^{A}	4.22 ±0.12^{H}	1.03 ±0.05^{AB}
		80	3.47 ±0.10^{A}	1.00 ±0.00^{A}	4.83 ±0.08^{H}	1.13 ±0.08^{A}	1.02 ±0.04^{A}	4.17 ±0.08^{GH}	1.02 ±0.04^{AB}
Yoghurt	3	60	4.98 ±0.04^{G}	1.02 ±0.04^{A}	3.80 ±0.13^{C}	1.12 ±0.08^{A}	1.00 ±0.00^{A}	3.78 ±0.08^{E}	1.02 ±0.04^{AB}
		80	4.80 ±0.1^{F}	1.02 ±0.04^{A}	4.55 ±0.10^{G}	1.07 ±0.05^{A}	1.02 ±0.04^{A}	3.25 ±0.05^{D}	1.02 ±0.04^{AB}
	6	60	3.80 ±0.09^{B}	1.02 ±0.04^{A}	3.95 ±0.05^{CD}	2.12 ±0.04^{E}	1.00 ±0.00^{A}	3.02 ±0.08^{AB}	1.70 ±0.09^{E}
		80	3.83 ±0.10^{BC}	1.00 ±0.00^{A}	3.82 ±0.04^{C}	2.10 ±0.09^{E}	1.00 ±0.00^{A}	3.17 ±0.10^{BCD}	1.62 ±0.08^{E}
Kefir	3	60	4.68 ±0.10^{F}	1.02 ±0.04^{A}	4.20 ±0.09^{E}	1.37 ±0.10^{B}	1.00 ±0.00^{A}	4.02 ±0.08^{FG}	1.22 ±0.08^{D}
		80	4.08 ±0.12^{DE}	1.00 ±0.00^{A}	4.41 ±0.10^{FG}	1.57 ±0.08^{C}	1.00 ±0.00^{A}	4.82 ±0.08^{J}	1.23 ±0.08^{D}
	6	60	4.03 ±0.08^{DE}	1.02 ±0.04^{A}	3.50 ±0.06^{B}	2.55 ±0.08^{F}	1.00 ±0.00^{A}	2.97 ±0.08^{A}	2.12 ±0.08^{G}
		80	3.77 ±0.08^{B}	1.03 ±0.05^{A}	3.78 ±0.04^{C}	1.98 ±0.10^{E}	1.00 ±0.00^{A}	3.20 ±0.09^{CD}	2.02 ±0.08^{G}
Butter milk	3	60	4.98 ±0.04^{G}	1.00 ±0.00^{A}	3.58 ±0.12^{B}	1.03 ±0.05^{A}	1.00 ±0.00^{A}	3.88 ±0.08^{EF}	1.12 ±0.08^{ABC}
		80	4.72 ±0.08^{F}	1.02 ±0.04^{A}	4.95 ±0.05^{H}	1.13 ±0.05^{A}	1.00 ±0.00^{A}	4.63 ±0.08^{I}	1.15 ±0.05^{BCD}
	6	60	4.18 ±0.08^{E}	1.03 ±0.05^{A}	4.23 ±0.05^{E}	1.82 ±0.08^{D}	1.00 ±0.00^{A}	3.05 ±0.05^{ABC}	1.87 ±0.08^{F}
		80	3.92 ±0.12^{BCD}	1.02 ±0.04^{A}	4.30 ±0.09^{EF}	1.55 ±0.08^{C}	1.00 ±0.00^{A}	2.98 ±0.08^{A}	1.28 ±0.08^{D}
		SEM	0.007	0.00106	0.007	0.0048	0.00065	0.007	0.0044
Effect of:									
Marinating type			***	NS	***	***	**	***	***
Marinating time			***	NS	***	***	NS	***	***
SV temperature			***	NS	***	***	NS	***	***
Marinating type x Marinating time			***	NS	***	***	NS	***	***
Marinating type x SV temperature			***	NS	***	***	NS	***	***

Marinating time x SV temperature	***	NS	***	***	NS	***	***
Marinating type x Marinating time x SV temperature	***	NS	***	***	NS	***	***

Mean± SD; (A, B, …) - different letters in column indicate significant differences between means for samples marinated in liquid fermented dairy products (FDP) and control sample (HSD test: $p \leq 0.05$); * = $p \leq 0.05$; ** = $p \leq 0.01$; *** = $p \leq 0.001$; NS - non-significant effect = $p > 0.05$.
